# Physical and Environmental Accessibility of Dental Services for Persons with Disabilities in Saudi Primary Health Care: A Cross-Sectional Study

**DOI:** 10.3390/healthcare14193225

**Published:** 2026-09-30

**Authors:** Faris Yahya I. Asiri, Maitham Abdullah Shabib, Salman Ahmed A. Alosayl, Mahdi Ali H. Al Shubaith, Ahmed Ibrahim T. Alsaqer, Mohammed Waleed Alyousef, Jasem Ahmad H. Alburaih, Estie Kruger

**Affiliations:** 1 Department of Preventive Dental Sciences, College of Dentistry, King Faisal University, Al Ahsa 31982, Saudi Arabia; 2 Dental Administration, Primary Healthcare, Al-Ahsa Health Cluster, Health Holding Company, Al-Ahsa 31982, Saudi Arabia; 3 Dental Intern, College of Dentistry, King Faisal University, Al-Ahsa 31982, Saudi Arabia; 4 Dental Services, Eastern Sector, Primary Healthcare, Al-Ahsa Health Cluster, Health Holding Company, Al-Ahsa 36421, Saudi Arabia; 5 International Research Collaboration—Health and Equity, School of Health and Clinical Sciences, The University of Western Australia, Perth, WA 6009, Australia; estie.kruger@uwa.edu.au

**Keywords:** persons with disabilities, health care accessibility, primary health care, special care dentistry, universal design, health equity, Saudi Arabia

## Abstract

**Background/Objectives**: Persons with disabilities (PWDs) may experience barriers when accessing oral health care, including those related to the physical and sensory environment of the dental-care setting. In this study we assessed the accessibility of primary health care centres (PHCs) providing dental services in Al-Ahsa, Saudi Arabia. **Methods**: We conducted a cross-sectional observational study across all 52 PHCs providing dental services in Al-Ahsa. We assessed facility-level accessibility using a structured checklist adapted from previous research and Saudi national accessibility standards. We classified accessibility features as appropriately installed, inappropriately installed, or not installed. **Results**: Basic physical accessibility was generally favourable, with appropriately installed main entrances in 92.3% of PHCs and entrance corridors in all PHCs. However, toilet seats and basin heights were appropriately installed in only 17.3% and 19.2% of PHCs, respectively, and warning and evacuation systems in only 13.5%. Braille blocks and transfer aids were absent in 98.1% of PHCs, whereas positioning supports and noise-reduction strategies were absent in all PHCs. None of the seven PHCs with dental operatories on upper floors had an appropriately installed elevator. No significant sector differences remained after correction for multiple comparisons. **Conclusions**: Basic physical access was generally favourable. However, many disability-specific accommodations were absent, and several existing features did not meet the predefined accessibility criteria. Regular assessments and targeted improvements to physical and environmental features in PHCs are needed to support inclusive oral health care for PWDs.

## 1. Introduction

Across diverse health-system settings globally, persons with disabilities (PWDs) continue to experience persistent barriers to accessing oral health care, including financial, provider-related, systemic, accessibility, and individual-level challenges. Structural obstacles include inaccessible entrances, narrow corridors, inadequate toilet facilities, and operatory-specific barriers such as insufficient wheelchair manoeuvring space and the absence of transfer-supportive dental chairs. Sensory barriers are also common and include the lack of Braille signage, tactile paving, and visual or auditory alarms, alongside environmental factors within dental settings such as bright operatory lighting and treatment-related noise that may compromise a comfortable and inclusive patient experience [1,2,3]. These barriers contribute to reduced utilisation of oral health care services, unmet treatment needs, and poor oral health outcomes among PWDs [3].

Evidence from diverse health care systems demonstrates that accessibility deficiencies are widespread in oral health care facilities, with essential accessibility features frequently absent or improperly installed [1,4]. Such findings underscore the need to adopt universal design principles to ensure that health care environments are accessible, inclusive, and responsive to the needs of PWDs [5].

In Saudi Arabia, the National Accessibility Standards [6] provide comprehensive requirements for public facilities, specifying design and functional criteria necessary to ensure accessibility for PWDs. Nevertheless, evidence regarding compliance with these standards in oral health care settings remains scarce. To date, limited local evidence is available on whether primary health care centres providing dental services in Al-Ahsa comply with national accessibility requirements, particularly in relation to dental treatment areas. Addressing this knowledge gap is particularly relevant in the context of Saudi Vision 2030 and the Health Sector Transformation Program, which emphasise facilitating access to healthcare services, improving the quality and efficiency of care, and addressing health inequalities [7].

National disability statistics have further highlighted the need for accessible health care environments. According to the 2016 national demographic survey, approximately 3.3% of Saudi citizens reported having a disability, equivalent to approximately one in every 30 citizens. Six regions exceeded the national rate, with prevalence ranging from 3437 to 4308 per 100,000; the Eastern Region reported 3506 per 100,000 [8]. A systematic review of studies conducted in Saudi Arabia identified poor oral health status and limited oral health care utilisation among PWDs, highlighting persistent challenges in meeting their oral health needs [9].

Dental services within the Saudi Ministry of Health are delivered through primary health care centres (PHCs), hospitals, dental complexes, and specialised dental centres. In 2023, the Ministry reported 3253 dental clinics nationwide, including 1639 located within PHCs [10]. Preventive and primary oral health care services in Saudi Arabia are commonly delivered through Ministry of Health PHCs, making PHCs an important setting for evaluating accessibility in community-based dental care. However, little is known about the extent to which these facilities provide an accessible environment for PWDs, particularly within dental treatment areas. Therefore, in this study we aimed to evaluate the accessibility of PHCs providing dental services in Al-Ahsa for PWDs by assessing structural, sensory, and dental-operatory accessibility features.

Using a standardised checklist adapted from international research and aligned with Saudi national accessibility standards [6], we sought to generate local evidence on accessibility compliance, identify priority deficiencies, and provide recommendations to support improved access to oral health care [7].

## 2. Materials and Methods

### 2.1. Study Design

In this study we employed a cross-sectional observational facility audit to evaluate accessibility within PHCs providing dental services in Al-Ahsa, Saudi Arabia. Data were collected between 27 January 2026 and 16 February 2026.

### 2.2. Study Setting and Population

We conducted this study in Ministry of Health PHCs located in Al-Ahsa, Eastern Province, Saudi Arabia. Within the Al-Ahsa PHC system, PHCs are geographically organised into four health sectors: Central, Northern, Southern, and Eastern. The study population comprised all PHCs providing dental services within the region. PHCs were eligible for inclusion if they provided active dental services at the time of data collection. A complete enumeration approach was adopted whereby all eligible PHCs providing dental services in Al-Ahsa (*n* = 52) were assessed. Accordingly, the study size was determined by complete enumeration rather than an a priori sample-size calculation. Accessibility assessments were completed in all 52 PHCs, yielding a response rate of 100%.

### 2.3. Instrument

The accessibility checklist was adapted from the instrument developed by Kim and Park [4], who assessed the installation status of accessibility features in dental institutions in South Korea. Their checklist primarily evaluated facility-level accessibility, including connecting facilities, internal facilities, hygiene facilities, and information facilities. To ensure contextual relevance and alignment with national accessibility standards, the checklist was modified based on the Universal Accessibility Built Environment Guidelines for the Kingdom of Saudi Arabia developed by the King Salman Center for Disability Research (KSCDR). Checklist items were reviewed and mapped against relevant KSCDR accessibility criteria, and wording was modified where necessary to reflect the Saudi primary health care context.

In addition, items relevant to the provision of dental care for individuals with disabilities were incorporated to assess accessibility within dental treatment areas. Based on recommendations in the special-care dentistry literature [2,11,12], this domain assessed wheelchair manoeuvring space, clear transfer space adjacent to the dental chair, removable or swing-away dental chair armrests, transfer aids (e.g., sliding boards or hoists), positioning supports (e.g., cushions or pillows), visual communication supports (e.g., communication boards, visual schedules, social stories), noise-reduction strategies (e.g., headphones or earplugs), and adjustable lighting for patients with sensory sensitivities.

The final checklist, reviewed by a panel of five experts from relevant dental disciplines for relevance and appropriateness within the study context, evaluated accessibility within primary health care centres providing dental services, encompassing both facility-level accessibility and accessibility within dental treatment areas. Consistent with Kim and Park [4], each item was classified as appropriately installed, inappropriately installed, or not installed according to predefined accessibility criteria.

An item was classified as appropriately installed when it met the relevant dimensional and functional criteria, inappropriately installed when it was present but did not meet those criteria, and not installed when it was absent. For example, the operational criteria included a corridor width of at least 1200 mm, a basin height of no more than 825 mm with at least 700 mm knee clearance, an approximate 1.5 m turning circle for wheelchair manoeuvring, and approximately 80 cm of clear space adjacent to the dental chair to facilitate side transfer. For items requiring dimensional verification, measurements were obtained using a laser distance meter (NF-271; Shenzhen Noyafa Electronic Co., Ltd., Shenzhen, China). Supplementary Table S1 provides the complete operational definitions and assessment criteria used to classify each accessibility feature, while Supplementary Form S1 presents the accessibility checklist used during data collection. A pilot phase involving five PHCs was conducted before the main data-collection phase. The pilot did not result in changes to the checklist items, response categories, dimensional thresholds, or classification criteria; instead, it clarified the interpretation and application of the existing operational definitions and standardised assessment and data-recording procedures among assessors. Accordingly, the five PHCs assessed during the pilot were retained in the final analysis because the same sampling frame and methodology were applied during the study.

### 2.4. Data Collection Procedure

Accessibility assessments were conducted through structured on-site observational audits using the standardised accessibility checklist. Four trained assessors participated in the data collection process. During each PHC visit, two assessors attended the facility and independently evaluated designated areas to ensure complete coverage of the centre. In PHCs with multiple dental operatories, assessment responsibilities were distributed between assessors to facilitate efficient data collection while maintaining standardised assessment procedures.

Accessibility features were evaluated through direct observation. For items requiring dimensional verification (e.g., corridor width, basin height, spatial clearance), measurements were obtained using a laser distance meter and cross-checked A conventional 5 m measuring tape (PowerLock, model STHT33158-8; Stanley Black & Decker, New Britain, CT, USA). to ensure measurement accuracy. Measurements were then classified according to the predefined accessibility criteria.

Data were recorded using standardised electronic data collection forms. Where needed, photographs were captured to support internal validation and reliability assessment, but these photographs were not used for public reporting. Each PHC was assigned a unique identification code to ensure confidentiality and maintain data integrity throughout the study.

Discrepancies between assessors were reviewed after independent data submission and resolved through discussion; unresolved cases were referred to the senior assessor or principal investigator.

### 2.5. Training, Calibration, and Reliability

Prior to data collection, all assessors underwent standardised training covering checklist interpretation, operational definitions of accessibility criteria, measurement techniques, and scoring procedures to ensure consistent application of the assessment tool.

A pilot phase involving five PHCs was conducted before the main data-collection phase. The five PHCs assessed during the pilot were retained in the final analysis because the same sampling frame and methodology were used throughout the study.

During the pilot phase, all four assessors independently evaluated the same facilities using the standardised checklist. Inter-rater reliability among the four assessors was assessed using Fleiss’ kappa across the three response categories. Agreement was excellent (κ = 0.889), with an overall observed agreement of 92.7%. The pilot did not result in changes to the checklist items, response categories, dimensional thresholds, or classification criteria; instead, it clarified the interpretation and application of the existing operational definitions and standardised assessment and data-recording procedures among assessors.

To assess whether scoring consistency was maintained during field implementation, inter-rater reliability was reassessed during the main data collection phase through concurrent evaluations conducted independently by all four assessors in three additional PHCs. Fleiss’ kappa was calculated. Agreement remained excellent (κ = 0.933), with an overall observed agreement of 95.77%, indicating sustained scoring consistency under field conditions. During pilot calibration and main-phase reliability assessments, assessors completed evaluations independently without discussion of scoring decisions prior to data submission.

### 2.6. Statistical Analysis

Data were analysed using IBM SPSS Statistics version 24 (IBM Corp., Armonk, NY, USA). Descriptive statistics were used to summarise the study variables. Means and standard deviations were calculated for continuous variables, and frequencies and percentages were calculated for categorical variables. Pearson’s chi-square test was used to examine associations between sector group and each accessibility item. When the assumptions of Pearson’s chi-square test were not met, Monte Carlo estimation was used. To account for 20 planned comparisons, the Bonferroni-adjusted significance level was set at α = 0.0025 (0.05/20). Cramér’s V was reported as the effect-size measure. Sector-level statistical tests were conducted as exploratory geographic comparisons within the Al-Ahsa PHC system.

The PHC was defined a priori as the unit of analysis. For PHCs containing multiple dental operatories, operatory-level observations were aggregated at the PHC level. For treatment-area items, a conservative worst-case approach was adopted whereby the lowest accessibility classification observed among operatories within the same PHC was assigned as the PHC-level score. This approach reflected whether accessibility was consistently available across operatories within the same PHC and ensured that barriers present in individual operatories were not obscured. No multivariable modelling was undertaken because the number of PHCs and the sparse or invariant categories for several accessibility items would not support stable multivariable estimates.

### 2.7. Ethical Considerations

The study protocol was initially reviewed by the Research Ethics Committee of King Faisal University, Saudi Arabia (Reference No. KFU-REC-2025-DEC-ETHICS3768), which determined that formal ethical approval was not required because the study involved a facility-based audit without direct human participant involvement.

Subsequently, ethical approval was obtained from the Institutional Review Board of King Fahd Hospital, Al-Hofuf, Al-Ahsa Health Cluster, Saudi Arabia (IRB Log No. 01-EP-2026; approval date: 11 January 2026). Administrative approval was obtained from the relevant health authorities prior to data collection.

No patient-level data were collected. All participating facilities were anonymised during data management, analysis, and reporting. Photographs captured during site assessments were used exclusively for internal validation and reliability assessment purposes. To protect the confidentiality of participating facilities, these photos were not publicly disseminated. No identifiable facility information was disclosed in the study findings.

## 3. Results

A total of 52 primary PHCs providing dental services were included in the study. The distribution of PHCs across the four sectors was balanced, with the Eastern sector accounting for the highest proportion (28.8%), followed by the Northern (26.9%), Southern (25.0%), and Central (19.2%) sectors.

Most dental operatories were located exclusively on the ground floor (45/52, 86.5%). Five PHCs (9.6%) had dental operatories on both the ground and upper floors, whereas two PHCs (3.8%) had dental operatories exclusively on the upper floor. Elevator accessibility was assessed in the seven PHCs with dental operatories located on upper floors. None had an appropriately installed elevator: Five had no elevator (71.4%), and two (28.6%) had inappropriately installed elevators.

The number of dental operatories per PHC ranged from 1 to 12, with a mean of 2.31 ± 2.68 operatories. Most PHCs had one dental operatory (33/52, 63.5%), while 10 (19.2%) had two operatories and nine (17.3%) had three or more operatories. (Table 1).

Table 2 presents the installation status of accessibility features in PHCs providing dental services in Al-Ahsa. Among the connecting facilities, access roads to the main entrance showed the highest level of appropriate installation (86.5%), followed by ramps (63.5%) and parking zones (55.8%). Regarding internal facilities, entrance corridors were appropriately installed in all PHCs (100.0%), whereas the main entrance/door was accessible in 92.3% of facilities. However, reception tables were inappropriately installed in more than half of the PHCs (53.8%).

For hygiene facilities, substantial deficiencies were observed. Appropriate installation of toilet seats and basin heights was reported in only 17.3% and 19.2% of PHCs, respectively, whereas more than 80% of facilities had inappropriate installations for both items.

Information facilities demonstrated the lowest levels of accessibility. Braille blocks were absent in 98.1% of PHCs, whereas guide and information systems were not installed in 88.5% of facilities. Warning and evacuation systems were appropriately installed in only 13.5% of PHCs, with 82.7% showing inappropriate installation.

With respect to operatory accessibility, clear transfer space adjacent to the dental chair was available in all PHCs (100.0%), whereas adequate wheelchair space and dental chairs with swing armrests were present in 65.4% and 71.2% of facilities, respectively. In contrast, transfer aids, positioning supports, visual communication supports, noise-reduction strategies, and sensory-adapted lighting were largely unavailable or inadequately installed.

Table 3 compares the installation status of facility-level accessibility features across the four PHC sectors. None of the accessibility features was significantly associated with a sector after Bonferroni correction (adjusted α = 0.0025). Descriptive variation was observed for parking zones, ramps, and access roads, with Cramér’s V estimates ranging from 0.265 to 0.353; however, the corresponding *p*-values did not meet the adjusted significance threshold.

Main entrances and entrance corridors showed generally high accessibility across sectors, whereas reception tables, hygiene facilities, and information facilities showed recurrent deficiencies. Although we report Cramér’s V estimates in Table 3, none of these sector comparisons was statistically significant after correction; therefore, the effect-size estimates should be interpreted cautiously.

Table 4 compares operatory accessibility features across the four sectors. No operatory feature was significantly associated with a sector after Bonferroni correction (adjusted α = 0.0025). Descriptive variation was observed in wheelchair manoeuvring space, whereas clear space adjacent to the dental chair was appropriately available in all PHCs, and dental chairs with swing armrests were appropriately installed in 60.0% to 76.9% of PHCs across sectors.

Five disability-specific accommodations were largely absent across all sectors. Transfer aids were unavailable in more than 92% of PHCs, positioning supports and noise-reduction strategies were absent in all facilities, and visual communication supports and sensory-adapted lighting were commonly absent or inappropriately installed. Despite descriptive differences among sectors, none of these comparisons reached the adjusted significance threshold.

## 4. Discussion

To our knowledge, the present study is the first in Saudi Arabia to comprehensively assess both facility-level and dental-operatory-specific accessibility features in PHCs providing dental services. In previous Saudi studies, researchers have identified oral health disparities among persons with disabilities, including low oral health-related quality of life, barriers to accessing oral health care, limited access to preventive dental services, and high unmet treatment needs [13]. The present study extends this evidence by directly assessing accessibility within both PHC facilities and dental treatment areas. This distinction is important because accessibility to a health care facility does not necessarily ensure accessibility to the environment in which dental care is provided.

Overall, the findings demonstrated a clear contrast between relatively favourable basic physical accessibility and substantial deficiencies in disability-specific accommodations. Fundamental features supporting entry and movement within PHCs were generally available, whereas important gaps remained in hygiene facilities, information and emergency systems, vertical accessibility, and dental-operatory accommodations. We observed accessibility deficiencies across all four sectors, although no sector differences reached statistical significance after correction for multiple comparisons. These findings indicate that physical access alone does not necessarily translate into an accessible dental-care environment responsive to the mobility, communication, sensory, and safety needs of PWDs.

External accessibility was generally favourable, particularly for access roads and main entrances, whereas accessible parking and ramps showed greater deficiencies. This pattern is broadly consistent with the findings of Kim and Park [4]; they reported that basic accessibility features, including access routes, ramps, and parking facilities, were more commonly available than several other disability-related accessibility features in dental institutions, although deficiencies in appropriate installation remained. Similarly, Asiri et al. [3] identified access-related barriers as an important component of the challenges that PWDs experience when seeking oral health care. The findings of the present study indicate that even when access to the facility is generally favourable, deficiencies at particular points along the access pathway may continue to restrict independent access for some PWDs. Therefore, regular assessment and appropriate modification of these features are important to support equitable access to dental services.

We observed a similar pattern within PHCs. Entrance corridors were consistently accessible, whereas reception and hygiene facilities demonstrated considerably greater deficiencies. Notably, inappropriate installation rather than complete absence accounted for most deficiencies in toilet seats and washbasins. This distinction has practical implications because some barriers may potentially be addressed by modifying existing infrastructure rather than requiring entirely new facilities. Vertical accessibility was also a concern. None of the seven PHCs with dental operatories on upper floors had an elevator meeting the predefined accessibility criteria. However, five of these PHCs also had ground-floor dental operatories, whereas two had dental operatories exclusively on an upper floor, where inadequate elevator accessibility represented a direct barrier to the dental treatment area. Kim and Park [4] similarly reported deficiencies in appropriately installed sanitary facilities in dental institutions. Yousef et al. [14] also identified accessibility gaps in an assessment of 56 PHCs in other parts of Eastern Saudi Arabia. However, their assessment addressed broader disability inclusion within PHCs, whereas in the present study we specifically evaluated PHCs providing dental services and extended the assessment to accessibility within dental treatment areas. Together, these findings emphasise that the presence of infrastructure alone should not be considered evidence of accessibility unless it is appropriately configured to meet relevant accessibility requirements.

Deficiencies were also evident in information, communication, sensory, and emergency accessibility accommodations. Braille blocks and guide and information systems were largely absent, whereas warning and evacuation systems were predominantly inappropriately installed. Kim and Park [4] similarly reported limited provision of Braille blocks and guide and information systems in dental institutions, while Pinto et al. [15] found that embossed or Braille information was rarely available in primary health care facilities. In contrast, they reported considerably better accessibility of alarm and evacuation systems than observed in the present study, suggesting that performance across individual accessibility domains may vary between health care contexts. Dougall and Fiske [12] highlighted the importance of tailored communication approaches for patients with hearing and visual impairments and the use of appropriate communication aids. These findings reinforce the need to consider information, communication, and emergency arrangements as integral components of accessibility rather than focusing exclusively on physical entry and mobility.

The dental-operatory findings further demonstrate the multidimensional nature of accessibility. Several fundamental spatial features were favourable, including clear space adjacent to dental chairs and, in many PHCs, adequate wheelchair manoeuvring space and dental chairs with swing armrests. However, disability-specific accommodations were substantially less available. Transfer aids were almost universally absent, positioning supports and noise-reduction strategies were absent in all assessed PHCs, and substantial deficiencies were observed in visual communication supports and lighting adjustments for sensory sensitivities. Makansi et al. [1] highlighted the influence of the physical and human dental environment on access to dental services among wheelchair users. Reynolds et al. [2] also reported that adaptations to visual, tactile, and auditory aspects of dental environments may positively influence anxiety-related responses among children and young adults with intellectual and developmental disabilities. Although in the present study we did not assess patient experiences or clinical outcomes, these findings indicate that accessibility within dental operatories should extend beyond spatial access to include appropriate transfer, positioning, communication, and sensory accommodations.

Taken together, the findings demonstrate that accessibility to dental services is multidimensional. Accessible entrances, corridors, and basic operatory space alone do not ensure an inclusive dental-care environment when hygiene facilities, communication and emergency systems, transfer equipment, and sensory accommodations remain inadequate. The absence of statistically significant sector differences after correction for multiple comparisons should not be interpreted as evidence of equivalent accessibility, particularly because deficiencies were identified across all four sectors. Improvement strategies should therefore consider accessibility across the Al-Ahsa PHC network rather than focusing on a single sector. Correcting improperly installed existing facilities may represent an achievable initial priority, alongside strengthening information and communication resources and providing appropriate positioning and sensory supports. Future renovation, construction, and procurement should follow Saudi national accessibility standards and be supported by regular accessibility audits to identify deficiencies and monitor improvement over time [6]. These priorities are consistent with the objectives of Saudi Vision 2030 and the Health Sector Transformation Program to improve access to and the quality of health care services [7], as well as the WHO Global Strategy and Action Plan on Oral Health 2023–2030, which emphasises equitable access to oral health care and its integration within primary health care and universal health coverage [16].

Priority should be given to correcting existing non-compliant hygiene and emergency features and providing commonly absent accommodations, including tactile guidance, transfer and positioning supports, visual communication aids, and noise-reduction measures.

Several limitations should be considered. First, we assessed the PHCs during a single study period; therefore, the findings represent facility conditions at the time of assessment and may not reflect subsequent modifications or improvements. Second, we restricted the study to Al-Ahsa, which limits the generalisability of the findings to other regions of Saudi Arabia and other health care settings. Third, we examined whether predefined accessibility features were present and appropriately installed but did not directly evaluate their usability or the experiences of PWDs. Accordingly, meeting an operational criterion should not necessarily be interpreted as evidence that a feature is accessible to all users. Fourth, because the number of dental operatories varied between PHCs, using the lowest operatory-level classification to derive the PHC-level classification may have increased the likelihood of a lower classification among centres with a greater number of operatories. Finally, the relatively small number of PHCs within each sector limited the suitability of the dataset for reliable multivariable modelling and, together with the conservative adjustment for multiple comparisons, may have limited the ability to detect sector differences.

Future research should extend facility-based accessibility audits to other regions of Saudi Arabia and across different health care sectors to determine whether similar patterns are observed elsewhere. Standards-based facility assessments should also be complemented by the perspectives and experiences of PWDs, caregivers, dental professionals, and facility managers because the presence and appropriate installation of an accessibility feature do not necessarily establish its usability in practice. Further validation of the adapted checklist across Saudi dental settings would strengthen its potential application in future accessibility assessments. Repeated audits could determine whether corrective actions and facility modifications result in measurable improvements over time. Future studies should also examine whether improvements in physical, communication, and sensory accessibility translate into frequent utilisation of dental services, positive patient experiences, few unmet treatment needs, and favourable oral-health outcomes among PWDs.

## 5. Conclusions

Basic structural access to PHCs providing dental services was generally favourable; however, important deficiencies were identified in hygiene facilities, information and emergency systems, vertical accessibility, and disability-specific dental-operatory accommodations. Regular accessibility assessments and adherence to national accessibility standards are recommended to address these deficiencies and support equitable access to primary oral health care for PWDs.

## Figures and Tables

**Table 1:** Demographic Characteristics.

Characteristics	*n* = 52 (%)
Sector	
Central	10 (19.2)
Northern	14 (26.9)
Southern	13 (25.0)
Eastern	15 (28.8)
Dental operatory location	
Ground floor only	45 (86.5)
Ground and upper floor	5 (9.6)
Upper floor only	2 (3.8)
Number of dental operatories (range = minimum − maximum)	1–12

**Table 2:** Installation Status of Connecting, Internal, Hygiene, and Information Facilities, and Dental-Operatory Accessibility Features.

Variable	Appropriate Installation *n* (%)	Inappropriate Installation *n* (%)	No Installation *n* (%)
Connecting facilities			
Access road to main entrance	45 (86.5)	5 (9.6)	2 (3.8)
Parking zone	29 (55.8)	18 (34.6)	5 (9.6)
Ramp	33 (63.5)	8 (15.4)	11(21.2)
Internal facilities			
Main entrance/door	48 (92.3)	4 (7.7)	0 (0)
Reception table	23 (44.2)	28 (53.8)	1 (1.9)
Entrance corridors	52 (100.0)	0 (0)	0 (0)
† Elevator	0 (0.0)	2 (28.6)	5 (71.4)
Hygiene facilities			
Toilet seat	9 (17.3)	42 (80.8)	1 (1.9)
Basin height	10 (19.2)	42 (80.8)	0 (0)
Information facilities			
Braille block	0 (0)	1 (1.9)	51 (98.1)
Guide and information system	0 (0)	6 (11.5)	46 (88.5)
Warning and evacuation system	7 (13.5)	43 (82.7)	2 (3.8)
Operatory accessibility			
Wheelchair manoeuvring space	34 (65.4)	18 (34.6)	0 (0)
Clear space adjacent to the dental chair to facilitate side transfer	52 (100.0)	0 (0)	0 (0)
Dental chair with swing armrest	37 (71.2)	1 (1.9)	14 (26.9)
Transfer aids	1 (1.9)	0 (0)	51 (98.1)
Availability of positioning support	0 (0)	0 (0)	52 (100.0)
Availability of visual communication support	5 (9.6)	11 (21.2)	36 (69.2)
Availability of lighting adjustments to accommodate sensory sensitivities	1 (1.9)	50 (96.2)	1 (1.9)
Availability of noise-reduction strategies	0 (0)	0 (0)	52 (100.0)

Note: Values represented are presented as counts *n* (row %). † Elevator accessibility was assessed only in PHCs with dental operatories located on upper floors (*n* = 7). PHCs with dental operatories located exclusively on the ground floor (*n* = 45) were considered not applicable and were excluded from this analysis. Percentages are based on applicable PHCs.

**Table 3:** Comparison of Accessibility-Feature Installation Status by Sector.

Variable	Central	Northern	Southern	Eastern	Effect Size µ	*p*-Value @
Connecting facilities						
Access road to main entrance						
Appropriate installation	9 (90)	13 (92.9)	9 (69.2)	14 (93.3)	0.265	0.294
Inappropriate installation	1(10.0)	1 (7.1)	2 (15.4)	1 (6.7)		
No installation	0 (0)	0 (0)	2 (15.4)	0 (0)		
Parking zone						
Appropriate installation	5 (50.0)	6 (42.9)	5 (38.5)	13 (86.7)	0.338	0.058
Inappropriate installation	5 (50.0)	6 (42.9)	5 (38.5)	2 (13.3)		
No installation	0 (0)	2 (14.3)	3 (23.1)	0 (0)		
Ramp						
Appropriate installation	8 (80.0)	8(57.1)	4 (30.8)	13 (86.7)	0.353	0.044
Inappropriate installation	1 (10.0)	2 (14.3)	5 (38.5)	0 (0)		
No installation	1 (10.0)	4 (28.6)	4 (30.8)	2 (13.3)		
Internal facilities						
Main entrance/door						
Appropriate installation	10 (100.0)	12 (85.7)	11 (84.6)	15 (100.0)	0.278	0.259
Inappropriate installation	0 (0)	2 (14.3)	2 (15.4)	0 (0)		
No installation	0 (0)	0 (0)	0 (0)	0 (0)		
Reception table						
Appropriate installation	5 (50.0)	8 (57.1)	4 (30.8)	6 (40.0)	0.215	0.572
Inappropriate installation	5 (50.0)	6 (42.9)	8 (61.5)	9 (60.0)		
No installation	0 (0)	0 (0)	1 (7.7)	0 (0)		
Entrance corridors						
Appropriate installation	10 (100.0)	14 (100.0)	13 (100.0)	15 (100.0)	-	-
Inappropriate installation	0 (0)	0 (0)	0 (0)	0 (0)		
No installation	0 (0)	0 (0)	0 (0)	0 (0)		
Elevator						
Appropriate installation	0 (0)	0 (0)	0 (0)	0 (0)	0.428	0.733
Inappropriate installation	1 (50.0)	1 (33.3)	0 (0)	0 (0)		
No installation	1 (50.0)	2 (66.7)	1 (100.0)	1 (100.0)		
Hygiene facilities						
Toilet seat						
Appropriate installation	2 (20.0)	3 (21.4)	1 (7.7)	3 (20.0)	0.195	0.681
Inappropriate installation	8 (80.0)	11 (78.6)	11 (84.6)	12 (80.0)		
No installation	0 (0)	0 (0)	1 (7.7)	0 (0)		
Basin height						
Appropriate installation	1 (10.0)	2 (14.3)	2 (15.4)	5 (33.3)	0.233	0.421
Inappropriate installation	9 (90.0)	12 (85.7)	11 (84.6)	10 (66.7)		
No installation	0 (0)	0 (0)	0 (0)	0 (0)		
Information facilities						
Braille block						
Appropriate installation	0 (0)	0 (0)	0 (0)	0 (0)	0.220	0.473
Inappropriate installation	0 (0)	0 (0)	0 (0)	1 (6.7)		
No installation	10 (100.0)	14 (100.0)	13 (100.0)	14 (93.3)		
Guide and information system						
Appropriate installation	0 (0)	0 (0)	0 (0)	0 (0)	0.321	0.148
Inappropriate installation	1 (10)	1 (7.1)	0 (0)	4 (26.7)		
No installation	9 (90.0)	13 (92.9)	13 (100.0)	11 (73.3)		
Warning and evacuation system						
Appropriate installation	3 (30.0)	2 (14.3)	2 (15.4)	0 (0)	0.327	0.084
Inappropriate installation	7 (70.0)	12 (85.7)	9 (69.2)	15 (100.0)		
No installation	0 (0)	0 (0)	2 (15.4)	0 (0)		

@ Pearson’s chi-square with Monte Carlo estimation and Bonferroni correction at α = 0.05/20 = 0.0025 µ Effect size calculated using Cramér’s V Values are presented as counts (column %).

**Table 4:** Comparison of Operatory Accessibility and Support by Sector Group.

Operatory Accessibility	Central	Northern	Southern	Eastern	Effect Size µ	*p*-Value @
Wheelchair manoeuvring space						
Appropriate installation	8 (80.0)	8 (57.1)	11 (84.6)	7 (46.7)	0.334	0.121
Inappropriate installation	2(20.0)	6 (42.9)	2 (15.4)	8 (53.3)		
No installation	0 (0)	0 (0)	0 (0)	0 (0)		
Clear space adjacent to the dental chair to facilitate side transfer						
Appropriate installation	10 (100.0)	14 (100.0)	13 (100.0)	15 (100.0)	-	-
Inappropriate installation	0 (0)	0 (0)	0 (0)	0 (0)		
No installation	0 (0)	0 (0)	0 (0)	0 (0)		
Dental chair with swing arm rest						
Appropriate installation	6 (60.0)	10 (71.4)	10 (76.9)	11 (73.3)	0.189	0.717
Inappropriate installation	0 (0)	0 (0)	0 (0)	1 (6.7)		
No installation	4 (40.0)	4 (28.6)	3 (23.1)	3 (20.0)		
Transfer aids						
Appropriate installation	0 (0)	0 (0)	1 (7.7)	0 (0)	0.243	0.383
Inappropriate installation	0 (0)	0 (0)	0 (0)	0 (0)		
No installation	10 (100.0)	14 (100.0)	12 (92.3)	15 (100.0)		
Availability of positioning support						
Appropriate installation	0 (0)	0 (0)	0 (0)	0 (0)	-	-
Inappropriate installation	0 (0)	0 (0)	0 (0)	0 (0)		
No installation	10 (100.0)	14 (100.0)	13 (100.0)	15 (100.0)		
Availability of visual communication support						
Appropriate installation	0 (0)	2 (14.3)	1 (7.7)	2 (13.3)	0.228	0.494
Inappropriate installation	3 (30.0)	2 (14.3)	1 (7.7)	5 (33.3)		
No installation	7 (70.0)	10 (71.4)	11 (84.6)	8 (53.3)		
Availability of lighting adjustments to accommodate sensory sensitivities						
Appropriate installation	0 (0)	1 (7.1)	0 (0)	0 (0)	0.260	0.320
Inappropriate installation	9 (90.0)	13 (92.9)	13 (100.0)	15 (100.0)		
No installation	1 (10.0)	0 (0)	0 (0)	0 (0)		
Availability of noise-reduction strategies						
Appropriate installation	0 (0)	0 (0)	0 (0)	0 (0)	-	-
Inappropriate installation	0 (0)	0 (0)	0 (0)	0 (0)		
No installation	10 (100.0)	14 (100.0)	13 (100.0)	15 (100.0)		

@ Pearson’s chi-square with Monte Carlo estimation and Bonferroni correction at α = 0.05/20 = 0.0025 µ Effect size calculated using Cramér’s V Values are presented as counts (column %).

## Data Availability

The raw data supporting the conclusions of this article will be made available by the authors on request.

## Supplementary Materials

The following supporting information can be downloaded at https://www.mdpi.com/article/10.3390/healthcare14193225/s1, Table S1. Operational definitions and criteria used to classify accessibility features; Form S1. Accessibility checklist used during data collection.
