# Supplementary material for: Physical and Environmental Accessibility of Dental Services for Persons with Disabilities in Saudi Primary Health Care: A Cross-Sectional Study
Source: Healthcare (Basel). 2026 Sep 30;14(19):3225. doi: 10.3390/healthcare14193225 (PMC13665206; doi:10.3390/healthcare14193225)
Supplement: Supplementary file 1 [file healthcare-14-03225-s001.zip › healthcare-4554577-supplementary.pdf]

The criteria below define the operational standards used to classify each accessibility feature during field assessment.

Supplementary Table S1. Operational definitions and criteria used to classify accessibility features.

Domain	Accessibility item	Assessment criterion
Connecting facilities	Access road to main entrance	Smooth, obstacle-free access route to the main entrance with a clear width of at least 1.2 m.
	Parking zone	An accessible parking space/zone is provided at the health centre.
	Ramp	Where required, an entrance ramp is provided with a minimum width of 95 cm, handrails, and a non-slip surface.
Internal facilities	Main entrance/door	Clear entrance width is at least 900 mm; an automatic door is preferred.
	Reception table	Reception counter/table height is less than 90 cm.
	Entrance corridors	Corridor width is at least 1200 mm and the route is free of obstacles.
	Elevator	Where an upper-floor dental clinic is present, an elevator is available; accessibility features such as Braille and visual/auditory signals are preferred.
Hygiene facilities	Accessible toilet	An accessible toilet is provided with an appropriate toilet seat, outward-opening door, and grab bar.
	Basin	Basin height is no more than 825 mm with at least 700 mm knee clearance.
Information and emergency facilities	Tactile/Braille block	Tactile paving/Braille blocks are provided along relevant routes and entrances.
	Guide and information system	Clear accessibility signage is provided, including Braille signs/maps where applicable.
	Warning and evacuation system	Visual and auditory alarms and accessible evacuation guidance are provided.
Dental-operatory accessibility	Wheelchair manoeuvring space	Sufficient space is available for wheelchair movement, including an approximately 1.5 m turning circle.
	Clear space adjacent to the dental chair to facilitate side transfer	Approximately 80 cm of clear space is available next to the dental chair to facilitate side transfer.
	Dental chair with swing armrest	The dental chair has a removable or swing-away armrest to facilitate transfer.
	Transfer aids	Transfer aids, such as a sliding board or hoist, are available in the clinic.
	Availability of positioning support	Positioning supports, such as cushions or pillows, are available to support the patient during care.
	Availability of visual communication support	Visual communication aids, such as pictures or boards, are available in the operatory.
	Availability of lighting adjustments to accommodate sensory sensitivities	Operatory lighting can be adjusted, for example using dimmable ceiling lights or an adjustable dental-unit light.
	Availability of noise-reduction strategies	Headphones with music or soothing sounds, or an equivalent noise-reduction strategy, are available to reduce dental noise.

Note. Elevator assessment applies only to PHCs with dental clinics located on an upper floor. Each item was classified as appropriately installed when it met the stated dimensional and functional criterion, inappropriately installed when present but not meeting the criterion, and not installed when absent.

Supplementary Form S1. Accessibility checklist used during data collection.

**PHC/Facility ID:**	**Assessment date:**
Sector:	Assessor(s):

**Domain**	**Accessibility item**	**Appropriate installation**	**Inappropriate installation**	**No installation**
Connecting facilities	Access road to main entrance	☐	☐	☐
	Parking zone	☐	☐	☐
	Ramp	☐	☐	☐
Internal facilities	Main entrance/door	☐	☐	☐
	Reception table	☐	☐	☐
	Entrance corridors	☐	☐	☐
	Elevator	☐	☐	☐
Hygiene facilities	Accessible toilet	☐	☐	☐
	Basin	☐	☐	☐
Information and emergency facilities	Tactile/Braille block	☐	☐	☐
	Guide and information system	☐	☐	☐
	Warning and evacuation system	☐	☐	☐
Dental-operatory accessibility	Wheelchair manoeuvring space	☐	☐	☐
	Clear space adjacent to the dental chair to facilitate side transfer	☐	☐	☐
	Dental chair with swing armrest	☐	☐	☐
	Transfer aids	☐	☐	☐
	Availability of positioning support	☐	☐	☐
	Availability of visual communication support	☐	☐	☐
	Availability of lighting adjustments to accommodate sensory sensitivities	☐	☐	☐
	Availability of noise-reduction strategies	☐	☐	☐

Classification: Appropriate installation = criterion met; Inappropriate installation = feature present but criterion not met; No installation = feature absent. Elevator assessment applies only to PHCs with dental clinics located on an upper floor.
